# Supplementary material for: Red Beetroot Fermentation with Different Microbial Consortia to Develop Foods with Improved Aromatic Features
Source: Foods. 2022 Oct 1;11(19):3055. doi: 10.3390/foods11193055 (PMC9562875; doi:10.3390/foods11193055)
Supplement: Supplementary file 1 [file foods-11-03055-s001.zip › foods-1865532-supplementary.pdf]

Red Beetrots fermentation with different microbial consortia to develop foods with improved aromatic features

Flavia Casciano [1§], Hannah Mayr[2§], Lorenzo Nissen [1,3*], Andreas Putti [2], Federica Zoli [2], Andrea Gianotti [1,3§], Lorenza Conterno [2§*]

[1] Department of Agricultural and Food Sciences (DISTAL), *Alma Mater Studiorum University of Bologna: Università di Bologna*, P.za Goidanich 60, 47521, Cesena, Italy.

[2] Laimburg Research Centre, Laimburg 6 – Pfatten/Vadena, 39040 Auer/Ora, BZ, Italy

[3] Interdepartmental Centre of Agri-Food Industrial Research (CIRI-AGRO), *Alma Mater Studiorum University of Bologna: Università di Bologna*, Via Q. Bucci 336, 47521 Cesena, Italy.

§ equally contributed

* Correspondence: lorenzo.nissen@unibo.it; lorenza.conterno@laimburg.it (LN) +39.0547.338134, (LC) +39.0471.969591

Supplementary Materials

Figure S1. Quantification heatmap of significant variables of the volatilome (ANOVA $P < 0.05$).

Figure S2. Texture analysis.

Figure S3. Significance of Spearman rank correlations.

Table S1. pH measurements.

Table S2. MANOVA categorical descriptors for the volatilome, categorized for the type of matrix.

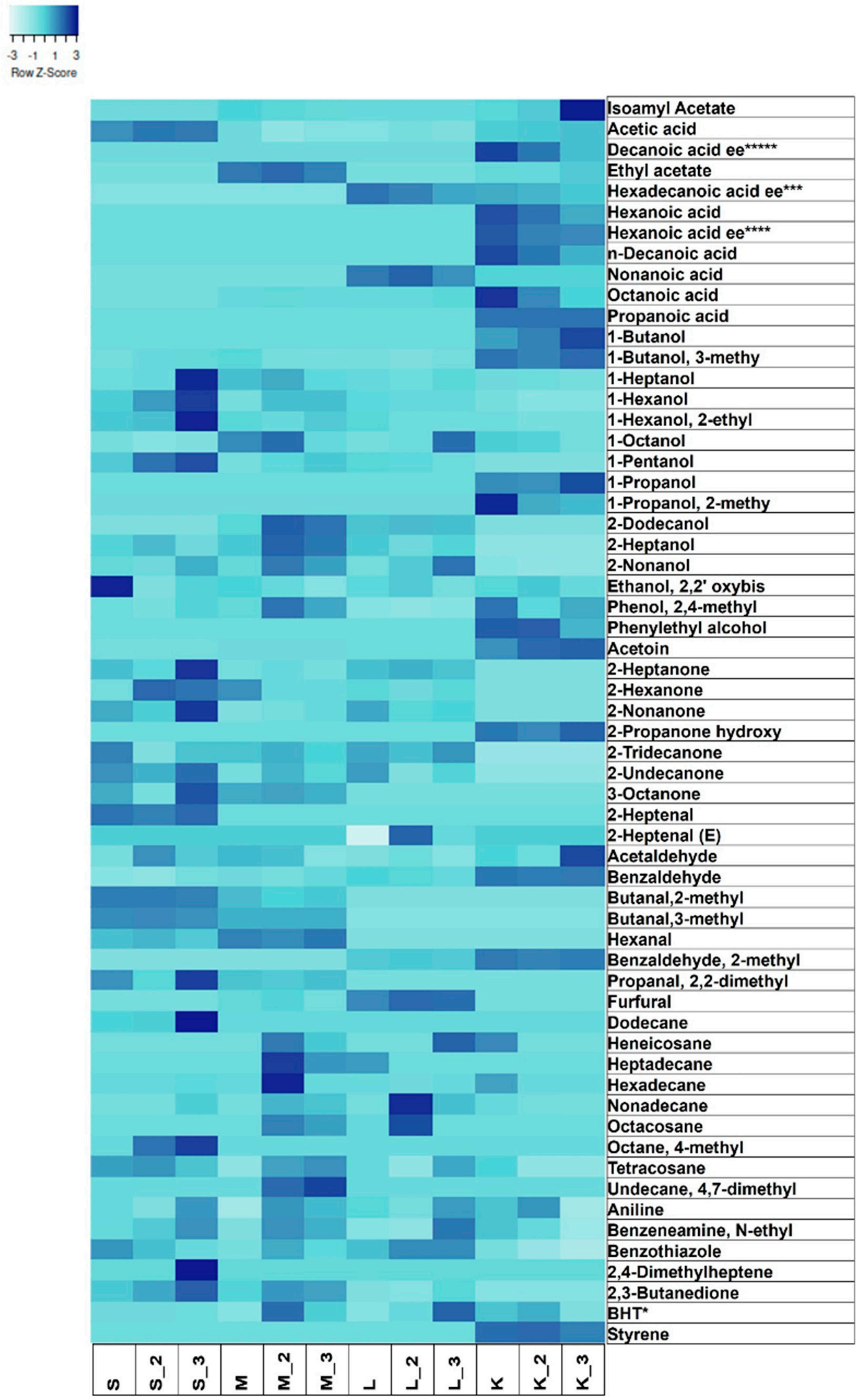

Figure S1. Quantification heatmap of significant variables of the volatilome (ANOVA $P < 0.05$).

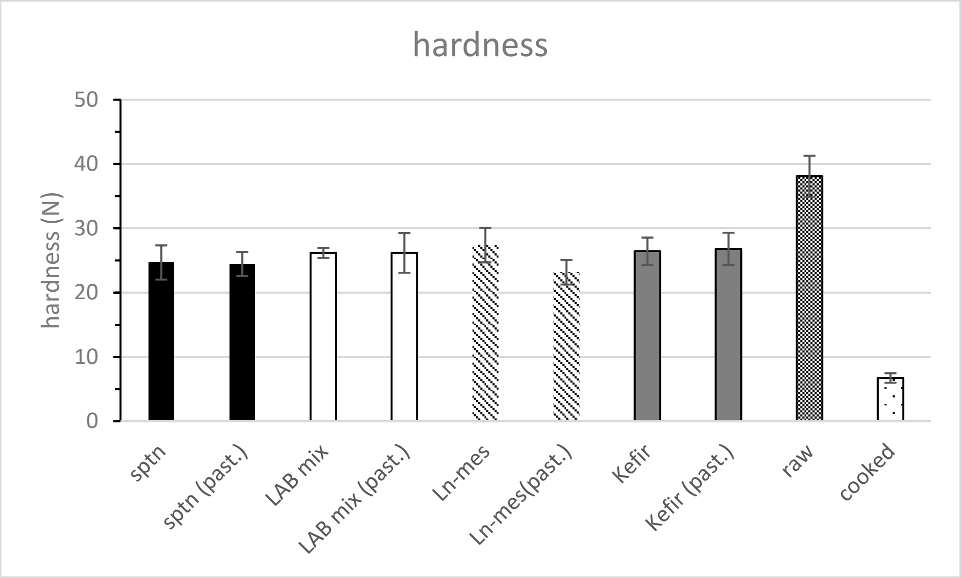

Figure S2: Texture results of beetroot samples fermented, fermented and pasteurized (past), compared to the raw and the cooked samples. Amounts reported are the average value of the replicates, the error bars representing the standard deviation "sptn": spontaneous fermentation; "LAB mix": fermentation after inoculum with mixed lactic acid bacteria starter culture; "Ln-mes": fermentation after inoculum with single-strain *Ln. mesenteroides* starter culture; "Kefir": fermentation after inoculum of LAB and yeasts water kefir starter. For this samples measurement was taken on five samples for each of the three fermentation replicates. For raw and cooked beetroot measurement was taken on five samples of one product.

Spearman Rank Order Correlations (Spreadsheet1)
MD pairwise deleted
Marked correlations are significant at p <,05000

Variable	Acetic acid	Decanoic acid, ethyl ester	Hexadecanoic acid, ethyl ester	Hexanoic acid	Hexanoic acid, ethyl ester	n-Decanoic acid	Octanoic acid
Batteri lattici	-0,756569	-0,542507	0,023435	-0,542507	-0,542507	-0,542507	-0,021393
Lieviti	0,013986	-0,247832	-0,132566	-0,247832	-0,247832	-0,247832	-0,138812
% sale	-0,388667	0,538242	0,552694	0,538242	0,538242	0,538242	0,613431
°Brix	-0,688938	-0,429077	-0,145026	-0,429077	-0,429077	-0,429077	-0,171749
delta pH	0,214414	0,618241	0,101910	0,618241	0,618241	0,618241	0,459786
SD + L-lattico (g/L)	0,419580	-0,211116	-0,538062	-0,211116	-0,211116	-0,211116	-0,651349
glucosio +fruttosio (g/L)	-0,027972	-0,734316	-0,826588	-0,734316	-0,734316	-0,734316	-0,872024
Durezza	-0,349650	0,110147	0,109172	0,110147	0,110147	0,110147	0,242031
gradimento odore	0,087566	-0,753993	-0,687427	-0,753993	-0,753993	-0,753993	-0,811156
gradimento sapore	-0,293706	-0,734316	-0,140364	-0,734316	-0,734316	-0,734316	-0,519655
gradimento complessivo	-0,452029	-0,742828	-0,329427	-0,742828	-0,742828	-0,742828	-0,440217
disposizione al consumo	0,000000	-0,543462	-0,696461	-0,543462	-0,543462	-0,543462	-0,571487

Variable	1-Butanol	1-Butanol, 3-methyl-	1-Hexanol	1-Pentanol	1-Propanol	1-Propanol, 2-methyl-	2-Dodecanol	2-Heptanol	Phenylethyl alcohol	2-Butanone, 3-hydroxy-	2,3-Butanedione
Batteri lattici	-0,616068	-0,623469	0,108582	0,091068	-0,570092	-0,516801	0,391769	0,412704	-0,542507	-0,360801	0,186951
Lieviti	-0,137684	-0,069930	-0,104895	-0,006993	-0,201937	-0,257948	0,224665	0,366206	-0,247832	-0,048520	0,063382
% sale	0,472717	-0,032092	-0,613309	-0,641835	0,482077	0,736135	0,133650	-0,344731	0,538242	0,618515	-0,682280
°Brix	-0,401395	-0,492098	0,210899	0,151144	-0,415236	-0,213305	0,451705	0,194691	-0,429077	-0,157587	0,269027
delta pH	0,655151	0,664333	-0,485068	-0,569428	0,622854	0,552084	0,179845	-0,173452	0,618241	0,632222	-0,304425
SD + L-lattico (g/L)	-0,174400	-0,111888	0,559441	0,699301	-0,165221	-0,149777	-0,183060	0,084509	-0,211116	-0,328445	0,352121
glucosio +fruttosio (g/L)	-0,752674	-0,461538	0,839161	0,916084	-0,734316	-0,665674	0,299553	0,711285	-0,734316	-0,731537	0,774667
Durezza	0,165221	-0,111888	-0,321678	-0,349650	0,211116	0,241307	0,257948	-0,140849	0,110147	0,526259	-0,387334
gradimento odore	-0,735603	-0,385290	0,802103	0,770579	-0,726408	-0,739776	0,020839	0,462088	-0,753993	-0,710386	0,719587
gradimento sapore	-0,752674	-0,706294	0,454545	0,482517	-0,761853	-0,590785	-0,062407	0,281697	-0,734316	-0,589708	0,330994
gradimento complessivo	-0,714797	-0,626434	0,370165	0,430673	-0,705453	-0,633156	0,455280	0,734810	-0,742828	-0,552805	0,422964
disposizione al consumo	-0,451349	-0,028070	0,610530	0,610530	-0,474377	-0,642963	0,313131	0,445232	-0,543462	-0,563690	0,826860

Variable	2-Nonanone	2-Undecanone	3-Octanone	Acetaldehyde	Benzaldehyde	Benzeneamine , N-ethyl-	Benzothiazole
Batteri lattici	0,080224	0,052911	0,102110	-0,479861	0,504416	0,262697	0,605955
Lieviti	-0,160168	-0,112679	-0,141456	-0,034965	-0,422546	-0,090909	-0,097902
% sale	-0,366607	-0,509915	-0,050914	-0,331615	0,405777	-0,217511	-0,199682
°Brix	0,334552	0,325664	0,533263	-0,358529	0,332744	0,186294	0,182779
delta pH	-0,763924	-0,555753	0,087832	0,590518	-0,569912	-0,196839	-0,826022
SD + L-lattico (g/L)	0,619315	0,549309	-0,029123	-0,027972	0,077467	-0,125874	0,027972
glucosio +fruttosio (g/L)	0,658467	0,760582	0,149777	-0,097902	0,105636	0,230769	0,349650
Durezza	-0,316776	-0,429588	0,128974	0,097902	0,288740	-0,545455	-0,216783
gradimento odore	0,732715	0,843045	0,058349	0,066550	-0,024692	0,178634	0,553416
gradimento sapore	0,772364	0,626776	-0,037444	-0,538462	0,380291	0,209790	0,818182
gradimento complessivo	0,338768	0,369197	0,021176	-0,380843	0,179222	0,242031	0,519655
disposizione al consumo	0,310746	0,572442	0,423771	0,192984	-0,367493	0,350879	-0,042106

Figure S3. Significance of Spearman rank correlations

Table S2. pH measurements during lactic fermentation expressed as means of three technical replicates plus standard deviation.

	1st day	13th day	20th day
**Spontaneous**	6.07 ± 0.26[a]	3.65 ± 0.04[b]	3.68 ± 0.04[b]
**LAB mix**	6.30 ± 0.21[a]	3.58 ± 0.04[b]	3.65 ± 0.05[b]
Ln mesenteroides	6.17 ± 0.15[a]	3.67 ± 0.04[b]	3.69 ± 0.03[b]
**Kefir**	6.17 ± 0.04[a]	3.55 ± 0.03[b]	3.63 ± 0.01[c]

[abc] Different letters indicate statistical significance by ANOVA followed by Tukey's HSD (Honestly Significant Difference) *post hoc* test ($P < 0.05$).

Table S2. MANOVA categorical descriptors for the volatilome, categorized for the type of matrix. % of contribution of VOCs descriptors significant among the food matrices.

**Volatile Organic Compounds**	**% of contribution of VOCs descriptors**				**MANOVA**
	**Spontaneous**	**Mix**	***Ln. mes****	**Kefir**	***P* value**
Isoamyl acetate	0.00	18.18	3.93	77.89	0.20373
Acetic acid	38.37[a]	16.99[b]	17.64[b]	27.00[ab]	0.00001
Decanoic acid, ethyl ester	0.00[b]	0.00[b]	0.00[b]	100.00[a]	0.00351
Ethyl acetate	1.92[c]	76.35[a]	1.45[c]	20.29[b]	<0.00001
Hexadecanoic acid, ethyl ester	0.00[c]	0.00[c]	61.03[a]	38.97[b]	0.00001
Hexanoic acid	0.00[b]	0.00[b]	0.00[b]	100.00[a]	0.00037
Hexanoic acid, ethyl ester	0.00[b]	0.00[b]	0.00[b]	100.00[a]	0.00001
n-Decanoic acid	0.00[b]	0.00[b]	0.00[b]	100.00[a]	0.00099
Nonanoic acid	0.00[c]	0.00[c]	76.09[a]	23.91[b]	<0.00001
Octanoic acid	0.00[c]	11.24[b]	13.71[b]	75.05[a]	0.02334
Propanoic acid	0.00[b]	0.00[b]	0.00[b]	100.00[a]	0.00051
1-Butanol	0.00[b]	0.00[b]	0.00[b]	100.00[a]	0.00021
1-Butanol, 3-methyl-	12.58[b]	10.50[b]	4.79[c]	72.13[a]	<0.00001
1-Heptanol	48.20	34.81	12.34	4.65	0.44823
1-Hexanol	50.27[a]	24.88[b]	18.48[b]	6.38[c]	0.03072
1-Hexanol, 2-ethyl-	63.70	20.69	12.42	3.20	0.09269
1-Octanol	7.28	42.73	28.78	21.21	0.18809
1-Pentanol	60.77[a]	18.63[b]	17.71[b]	2.89[c]	0.01085
1-Propanol	0.00	0.00	0.00	100.00[a]	0.00007
1-Propanol, 2-methyl-	0.00[b]	0.00[b]	0.00[b]	100.00[a]	0.02325
2-Dodecanol	0.00[c]	64.86[a]	35.14[b]	0.00[c]	0.00638
2-Heptanol	23.73[b]	53.78[a]	22.49[b]	0.00[c]	0.00422
2-Nonanol	24.88	35.69	31.26	8.17	0.09163
Ethanol, 2,2'-oxybis-	38.14	17.53	21.03	23.29	0.61613
Phenol, 2,4-bis(1,1-dme)-*	20.98[ab]	31.99[a]	14.91[b]	32.13[a]	0.03854
Phenylethyl alcohol	0.00[b]	0.00[b]	0.00[b]	100.00[a]	0.00080
2-Butanone, 3-hydroxy-	0.00[c]	4.81[b]	2.09[b]	93.10[a]	<0.00001
2-Heptanone	50.65	9.42	36.40	3.52	0.07820
2-Hexanone	53.94	28.75	17.31	0.00	0.13754
2-Nonanone	61.88 [a]	5.84[c]	32.28[b]	0.00[d]	0.03272
2-Propanone, 1-hydroxy-	0.00[b]	0.00[b]	0.00[b]	100.00[a]	<0.00001
2-Tridecanone	30.59[a]	30.66[a]	38.75[a]	0.00[b]	0.01377

2-Undecanone	50.14[a]	23.55[b]	26.31[b]	0.00[c]	0.01097
2-Octanone	49.51[a]	50.49[a]	0.00[b]	0.00[b]	0.04905
2,3-Butanedione	49.01	50.99	0.00	0.00	0.05003
2-Heptenal	100.00[a]	0.00[b]	0.00[b]	0.00[b]	<0.00001
2-Heptenal, (E)-	0.00	0.00	100.00	0.00	0.94485
Acetaldehyde	29.72	24.04	8.41	37.83	0.41336
Benzaldehyde	10.39[b]	17.23[b]	22.76[ab]	49.62[a]	<0.00001
Butanal, 2-methyl-	68.26[a]	31.74[b]	0.00[c]	0.00[c]	<0.00001
Butanal, 3-methyl-	57.49[a]	42.51[a]	0.00[b]	0.00[b]	<0.00001
Hexanal	34.94[b]	65.06[a]	0.00[c]	0.00[c]	<0.00001
Benzaldehyde, 2-methyl-	0.00[c]	0.00[c]	30.52[b]	69.48[a]	<0.00001
Propanal, 2,2-dimethyl-	66.08[a]	33.92[b]	0.00[c]	0.00[c]	0.01803
Furfural	0.00[c]	12.91[b]	87.09[a]	0.00[c]	<0.00001

[abcd] Different letters indicate statistical significance by MANOVA followed by Tukey's HSD (Honestly Significant Difference) *post hoc* test ($P < 0.05$). **Ln. mes.* = *Leuconostoc mesenteroides*
